# Continuous Selective Intra-Arterial Application of Nimodipine in Refractory Cerebral Vasospasm due to Aneurysmal Subarachnoid Hemorrhage

**DOI:** 10.1155/2014/970741

**Published:** 2014-01-16

**Authors:** Stephanie Ott, Sheila Jedlicka, Stefan Wolf, Mozes Peter, Christine Pudenz, Patrick Merker, Ludwig Schürer, Christianto Benjamin Lumenta

**Affiliations:** ^1^Department of Neurosurgery, Academic Teaching Hospital Munich-Bogenhausen, Technical University of Munich, Englschalkinger Straße 77, 81925 Munich, Germany; ^2^Department of Neurosurgery, Charité University Hospital, Augustenburger Platz 1, 13353 Berlin, Germany; ^3^Department of Radiology, Academic Teaching Hospital Munich-Bogenhausen, Technical University of Munich, Englschalkinger Straße 77, 81925 Munich, Germany; ^4^Department of Neurosurgery, Academic Teaching Hospital Munich-Schwabing, Ludwig Maximilian University of Munich, Kölner Platz 1, 80804 Munich, Germany

## Abstract

*Background*. Cerebral vasospasm is one of the leading courses for disability in aneurysmal subarachnoid hemorrhage. Effective treatment of vasospasm is therefore one of the main priorities for these patients. We report about a case series of continuous intra-arterial infusion of the calcium channel antagonist nimodipine for 1–5 days on the intensive care unit. *Methods*. In thirty patients with aneurysmal subarachnoid hemorrhage and refractory vasospasm continuous infusion of nimodipine was started on the neurosurgical intensive care unit. The effect of nimodipine on brain perfusion, cerebral blood flow, brain tissue oxygenation, and blood flow velocity in cerebral arteries was monitored. *Results*. Based on Hunt & Hess grades on admission, 83% survived in a good clinical condition and 23% recovered without an apparent neurological deficit. Persistent ischemic areas were seen in 100% of patients with GOS 1–3 and in 69% of GOS 4-5 patients. Regional cerebral blood flow and computed tomography perfusion scanning showed adequate correlation with nimodipine application and angiographic vasospasm. Transcranial Doppler turned out to be unreliable with interexaminer variance and failure of detecting vasospasm or missing the improvement. *Conclusion*. Local continuous intra-arterial nimodipine treatment for refractory cerebral vasospasm after aSAH can be recommended as a low-risk treatment in addition to established endovascular therapies.

## 1. Introduction

The incidence of aneurysmal subarachnoid hemorrhage is located between 6 and 8 people out of every 100,000 each year [1, 2]. 46% [1] to over 50% [3] of patients die within the first two weeks of their aSAH. Medical care is not reached by about 10–15% [4]. Complications such as neurogenic pulmonary edema or neurogenic stunned myocardium kill 25% of the treated patients [5]. Vasospasm is one of the main causes for prolonged neurologic deficit in patients who reach either neurosurgical or endovascular treatment for the aneurysm. 7% die of vasospasm and another 7% develop severe neurologic deficit [6]. Treatment of vasospasm is therefore one of the main priorities for these patients.

Mechanical endovascular interventions such as balloon angioplasty or stenting are options for vasospasm of the great vessels; however, the distally located smaller vessels cannot be reached by the neuroradiologist and therefore need to be treated with pharmacologic agents [7]. Amrinone [8], l-arginine [9], colforsin daropate hydrochloride [10], papaverine [11], verapamil [12], nicardipine [13], and nimodipine [14, 15] have been tested for their effect on vasospasm, but none have shown significant difference to the others.

The pharmacological effect on the vessels is in some cases limited to the period of infusion—either the procedure has to be repeated [16] or the agent has to be applied continuously for a few days until there is no more evidence of vasospasm in neuromonitoring and angiography.

At our hospital all patients with aSAH are treated with intravenous or oral nimodipine from the day of admission for at least 21 days. Patients with detected vasospasm on angiography receive intra-arterial nimodipine as a bolus application in the angiography unit. Side effects as rapid increase in ICP, thrombocytopenia, seizures, transient neurologic deficits (mydriasis and brainstem depression), monocular blindness, and paradoxical worsening of vasospasm as it is reported for intra-arterial papaverine [16–19] were not seen.

We want to report our experience with continuous selective intra-arterial infusion of nimodipine via a catheter in the internal carotid artery in refractory cerebral vasospasm.

## 2. Materials and Methods

### 2.1. Patient Population

From March 2006 until July 2013, 27 patients, who suffered aneurysmal subarachnoid hemorrhage and developed cerebral vasospasm refractory to standard hyperdynamic and endovascular therapy, were respectfully treated with locally infused nimodipine via catheter in the internal carotid artery for minimum of one day at the neurosurgical intensive care unit at the Academic Teaching Hospital of the Technical University of Munich, Klinikum Munich-Bogenhausen. Two patients with clipped incidental median cerebral artery (MCA)-aneurysms, who developed refractory vasospasm seven and five days after the operation, were included as well. One patient was treated at the interdisciplinary surgical intensive care unit at the academic teaching hospital of the Ludwig Maximilian University Munich, Klinikum Munich-Schwabing. Data from 2006 to September 2009 were analyzed retrospectively. During that period of time, nine patients were treated by continuous infusion of intra-arterial nimodipine. All patients, who were treated from October 2009 to July 2013, were included in the study prospectively. No patient was excluded. Randomization was not performed.

### 2.2. Management of Subarachnoid Hemorrhage

In 26 patients, the aneurysm, causing the hemorrhage, was clipped. There was one reclipping of a right-sided media aneurysm located at the bifurcation. Vasospasm occurred nine days after the second operation. In one case, there was only a distal median cerebral artery (segment 1 (M1)-) aneurysm, diagnosed in the computed tomography angiography, which was clipped. After rebleeding, a digital subtraction angiography was performed, showing a second aneurysm located at the supraclinoidal internal carotid artery (ICA), which was coiled in the same session. The MCA aneurysm was shown to be completely obstructed by the clip. We therefore proposed that the ICA aneurysm had been ruptured initially. One patient with a communicating artery aneurysm died before treatment of the aneurysm. We recorded only two patients who were exclusively treated by coiling.

Detection of vasospasm depended on the initial Hunt & Hess grade. Patients grouped from H&H 0–2 usually developed either a focal neurologic deficit, for example, speech disturbance and hemiparesis, or loss of consciousness. Those who were classified as Hunt & Hess 3 or higher on admission were monitored with regional cerebral blood flow (rCBF) with the Bowman system (Hemedex Inc., Cambridge, MA, USA) and local tissue oxygenation with Licox probes (Integra Neuroscience, Andover, UK). External ventricular drainages were placed in the right lateral ventricle to measure intracranial pressure and drain cerebrospinal fluid (CSF) when necessary. If Hunt & Hess 0–2 patients lost consciousness due to vasospasm or other reason they were monitored equally to the Hunt & Hess 3+ patients. When angiography was performed prior to placing rCBF and ptiO2 probes, they were located in an area supplied by a vasospastic vessel. Depending on CSF circulation an external ventricular drainage or a parenchymal probe was placed.

Data were monitored by ICUpilot (CMA, Solna, Sweden) with sampling frequency of 1/min. Depth of anesthesia could be supervised with bispectral index (BIS) (Covidien Healthcare).

Transcranial Doppler measurements were taken daily or every second day.

### 2.3. Management of Vasospasm

After clipping or coiling of the aneurysm systolic blood pressure was elevated to 150 mmHg. When detecting vasospasm we started standard triple H therapy with hypertension, hypervolemia, and hemodilution.

In cases of neurologic or neuromonitoring parameter deterioration, a CAT scan in combination with perfusion CT was performed to rule out causes other than vasospasm and to detect areas of reduced or delayed cerebral perfusion and blood flow or manifested ischemic areas marked as infarctions already. The perfusion scan should represent all territories of the three main cerebral arteries, namely, the anterior, middle, and posterior cerebral artery, and is therefore located at the upper basal ganglia as an axial slice.

Digital subtraction angiography was performed in general anesthesia without exception. Every patient with vasospasm was treated with a thirty-minute lasting intra-arterial infusion of 10 mL (=2 mg) nimodipine via the catheter in the internal carotid artery. If postinfusion angiography showed complete recurrence of vasospasm the catheter was taken out. When vasospasm persisted after short-time local intraarterial nimodipine infusion, the catheter was left in place and continuous intra-arterial nimodipine infusion was started on the intensive care unit. During the period of intra-arterial nimodipine application, all patients who had lost consciousness before angiography were sedated and mechanically ventilated during the period of intra-arterial nimodipine application on the ICU. Patients, who suffered from specific focal neurologic deficit without impairment of consciousness, were awakened after initial angiography with catheter placement in order to monitor either the paresis or speech deficit. In cases of elevated intracranial pressure (ICP), all standard conservative treatment options were exhausted before surgery was performed. Three of our thirty patients underwent decompressive hemicraniectomy. One had developed infarction of the right cerebellar hemisphere due to vasospasm of the posterior inferior cerebellar artery (PICA) and therefore had to be craniectomied suboccipitally.

**Figure 1:**
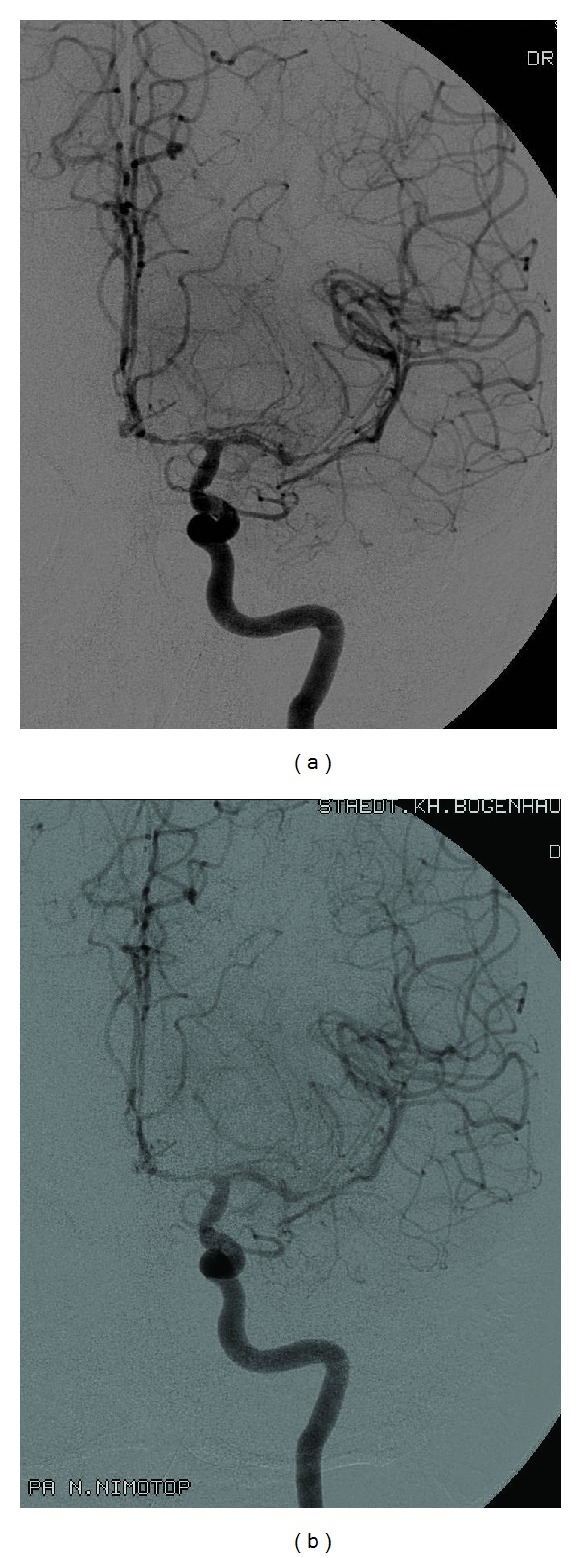
DSA before and after nimodipine application at the angiography unit. Digital subtraction angiography of the left internal carotid artery before (a) and after (b) nimodipine application in the angiography unit. It shows slight improvement of vasospasm after nimodipine infusion. Intra-arterial infusion of the calcium channel antagonist was continued on the neurosurgical intensive care unit.

For angiography, four or five French introducer sheaths with a microcatheter were used. Before administration of nimodipine, digital subtraction angiography was performed by contrast application into both internal carotid arteries. After identification of vasospastic vessels (see Figure 1), 0.2–0.4 mg nimodipine (1-2 mL nimodipine solution) was administered via the catheter in the right or left ICA, depending on the side of leading vasospasm, as a bolus. A 30-minute infusion containing 2 mg nimodipine (10 mL nimodipine solution) was then administered. To visualize the effect of nimodipine on the vessels, a further angiography was performed. Patients where no vasospasm could be noticed on the second angiography had their catheters removed. These patients included in our study showed slight improvement of vasospasm but not complete normalization of vessel diameter and configuration. The catheter was fixed with the tip in the extracranial internal carotid artery for further continuous nimodipine infusion at the ICU. In our retrospective analyzed patient data, four patients were treated with a 30-minute infusion of 10 mL (2 mg) nimodipine every 8 hours for 2–5 days. In five of our retrospective patient data and all of the prospective treated patients, the protocol was changed to an infusion rate of 5 mL/h (1 mg/h) applied continuously. Via a second line, which was connected to the catheter with the tip placed in the external iliac artery, 10.000 IU/24 h of heparin was given to prevent clotting of the introducer sheaths with the microcatheter and avoid thrombosis related to the nimodipine catheter tip in the internal carotid artery.

Depending on the neuromonitoring parameters or, in wake patients, their improvement of symptoms, intra-arterial nimodipine infusion was stopped after 1–5 days. In most cases before catheter removal, another angiographic control was performed to observe the therapeutic effect on the vessels in correlation with clinical improvement. On the other hand, to avoid missing persistent vasospasm, that requires further treatment. Nevertheless, we missed the final angiography in a few cases because of weekend or public holiday on the day of catheter removal.

## 3. Results

### 3.1. Baseline Characteristics

Patient data are listed in Table 1. Mean patient age was 49.77 ± 10.67 standard deviation (SD) years. The youngest was a 27-year-old woman and the oldest a 76-year-old man. Mean Hunt & Hess grade was 3.07 ± 1.41 with 27% of the patients being graded Hunt & Hess 2. Hunt & Hess 3 was documented in 23% and 20% were Hunt & Hess 4 and 5 each.

Mean onset of continuous intra-arterial nimodipine application was day 9.19 ± 3.31.

### 3.2. Treatment Results

Mean outcome of all patients was GOS 3.27 ± 1.46. From 19 patients with Hunt & Hess grade 3 and higher four patients died (GOS 1), three were in a persistent vegetative state (GOS 2), and six had severe disability with dependence on support (GOS 3). One case received GOS 4 with minor disability and five patients left the hospital without apparent deficits (GOS 5). Persistent ischemic areas were seen in 100% of patients with GOS 1–3 and in 69% of GOS 4-5 patients.

In four patients vasospasm was shown to be refractory to local nimodipine administration. They died from vasospastic induced major cerebral infarcts. Another patient died from other reason than vasospasm, which was angiographically cured. Overall results from continuous intra-arterial nimodipine infusion were promising. 26 patients improved from angiographically documented refractory vasospasm. Anyway from the 25 survivors only 4 (16%) did not have ischemic brain tissue areas at discharge. All of them reached GOS 5. 17 (68%) patients showed minor cerebral infarcts, which were in multiple locations in 6 (24%) cases. Depending on eloquence of the ischemic brain tissue, patients varied between GOS 3 and 5. Patients having major cerebral infarcts, which occurred in 4 of the 25, left the hospital in a persistent vegetative state (GOS 2).

After having finished the continuous intra-arterial nimodipine therapy with resolved vasospasm on angiography, four patients relapsed in having vasospasm and needed further intra-arterial nimodipine treatment. One patient even had to undergo three cycles before he recovered and ended up without any neurologic deficit on discharge. The 56-year-old female patient with a noted treatment onset on day 20 after bleeding had had two sessions of intra-arterial nimodipine bolus applications at the angiography unit of Klinikum Schwabing.

11 patients were graded Hunt & Hess equal or better than 2. One of those patients died of refractory cerebral vasospasm even before treatment of the aneurysm. Two had severe disabilities and one is in a persistent vegetative state. Of the five patients, who died during hospital stay, four developed limiting infarctions due to refractory cerebral vasospasm and one died of sepsis three days after ICA-catheter removal. Microbiological tests found the catheter tip to be sterile. Since the catheter is at least 50 cm in length and only the tip was sent away for tests, the result cannot be representative of the whole catheter.

On CT angiography, we saw thrombotic vessel wall adhesions within the extracranial common or internal carotid artery in three patients. As all of these patients had cerebral infarctions, an additional embolic insult cannot be excluded. The ischemic areas anyway were all limited to territories of vasospastic vessels.

Thirteen patients survived in a good clinical condition (Glasgow outcome scale (GOS 4 and 5)). Five patients classified as Hunt & Hess 3 and 4 on admission left the hospital without apparent deficits (GOS 5).

**Figure 2:**
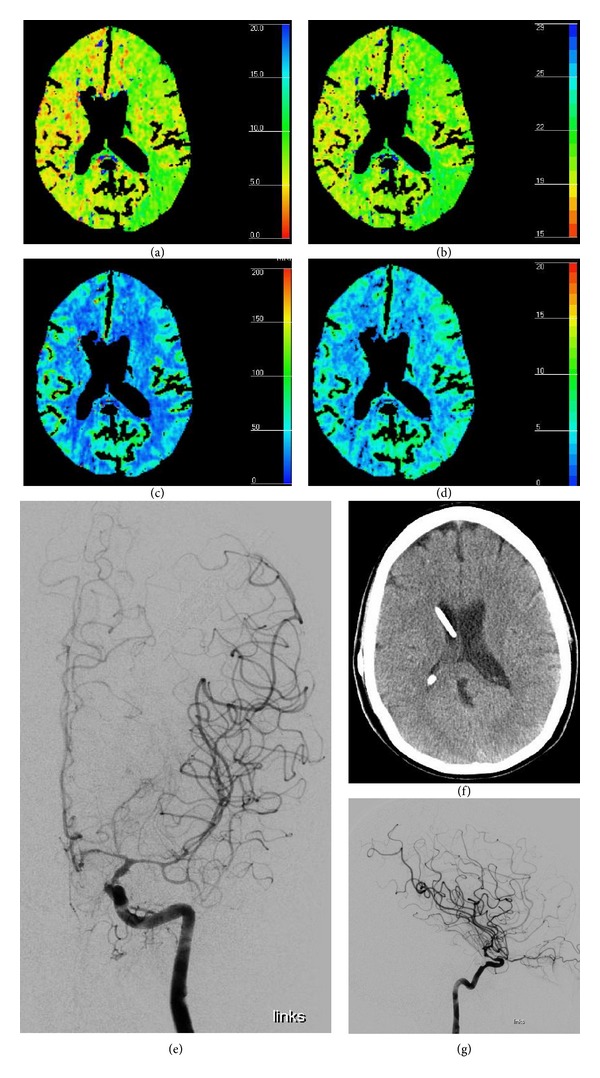
Perfusion CT correlating with DSA. CT perfusion with MTT (sec) (a), TTP (sec) (b), CBF (mL/100 g/min) (c), and CBV (mL/100 g) (d) showing circumscribed perfusion deficit on the territory of the left MCA. No evidence of infarction as shown on native CT scan (f). Digital subtraction angiography (e, g) of the left ICA from the same patient on the same day showing vasospasm of the left ICA above the ophthalmic artery of the median and anterior cerebral artery.

Addressing the neuromonitoring modalities, we noticed the greatest effects of intra-arterial nimodipine infusion to rCBF parameters; see Table 2. ptiO2 values showed prompt but lower alterations; see Table 3. Changes of intracranial pressure were no indicator for vasospasm. Perfusion CT scans showed vasospasm related changes in 90% of our cases and showed improvement correlating with angiographic results and clinical symptoms; see Figure 2. Data from transcranial Doppler were shown not to be reliable for exclusion or detection of vasospasm; see Table 4. For patient 1 improvement of vasospasm is not shown and in patients 4 and 9 vasospasm is not detected at all. Worsening of vasospasm is missed in patients 25 and 30; only patient 10 showed adequate changes in blood flow velocity.

**Figure 3:**
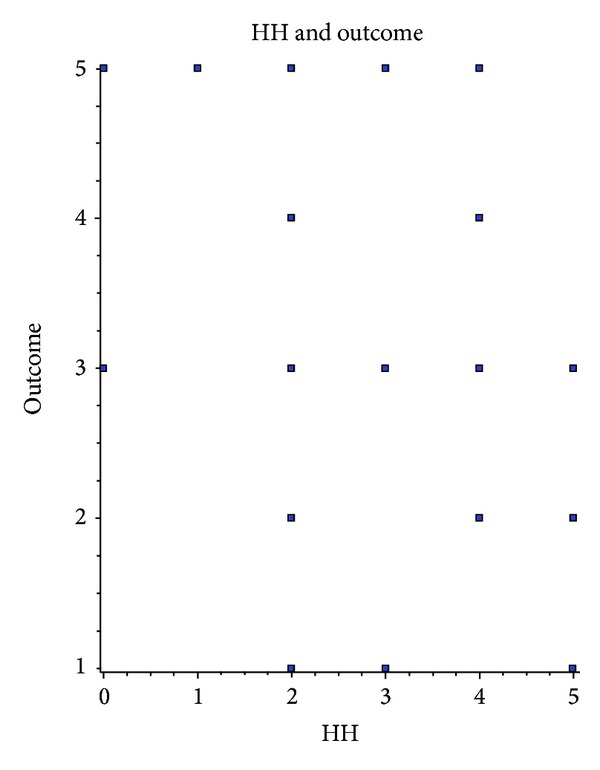
Correlation of H&H grade and patient outcome. Spearman rank test shows inverted correlation of Hunt & Hess grade on admission and outcome (GOS) on discharge.

**Figure 4:**
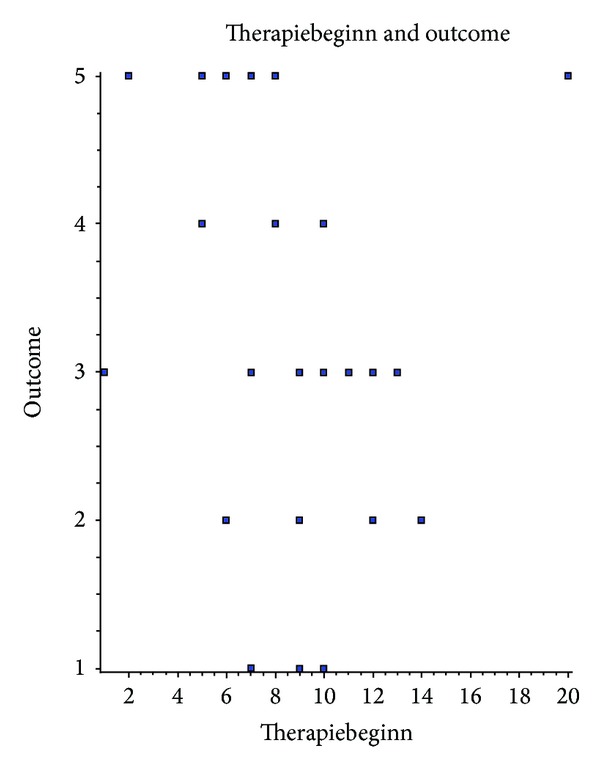
Correlation of onset of selective continuous intra-arterial nimodipine infusion and patient outcome. Spearman rank test shows that inverted correlation of the day nimodipine infusion was started with the outcome (GOS) on discharge.

We could not detect correlation between patient age and duration of selective intra-arterial nimodipine application (Spearman *r* = −0.2290 (corrected for ties), 95% confidence interval: −0.5522 to 0.1541, two-tailed *P* value is 0.2236) or outcome (Spearman *r* = −0.1642 (corrected for ties), 95% confidence interval: −0.5036 to 0.2192, two-tailed *P* value is 0.3860, considered not significant). Results were the same for correlation of sex and other parameters (Fisher's exact test and Spearman correlation test). Hunt & Hess did not predict the duration of therapy (Spearman *r* = 0.07569 (corrected for ties), 95% confidence interval: −0.3028 to 0.4336, two-tailed *P* value is 0.6910), but the Spearman rank correlation test showed significant difference in outcome (Spearman *r* = −0.4353 (corrected for ties), 95% confidence interval: −0.6936 to −0.07778, two-tailed *P* value is 0.0162); see Figure 3. The onset of continuous intra-arterial nimodipine treatment significantly correlates with the outcome (GOS) (Spearman *r* = −0.4070 (corrected for ties), 95% confidence interval: −0.675 to −0.04351, two-tailed *P* value is 0.0256); see Figure 4.

**Figure 5:**
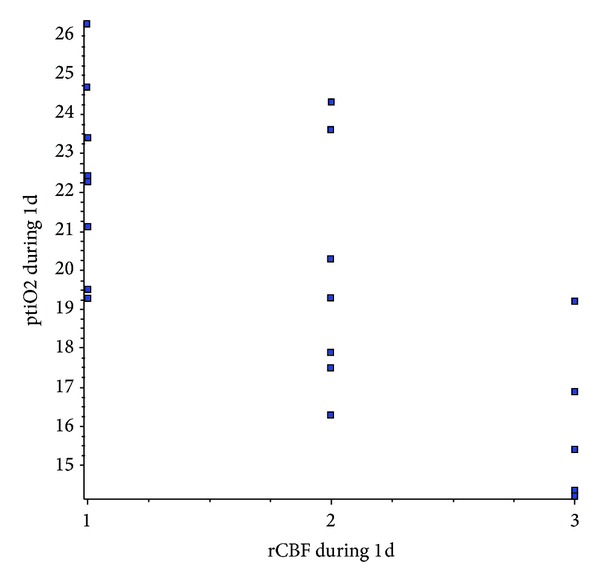
Linear correlation test shows extremely high significance of ptiO2 and rCBF values during (day 1) selective continuous intra-arterial nimodipine infusion.

Linear correlation of rCBF and ptiO2 before intra-arterial nimodipine treatment was not significant (correlation coefficient (*r*) = −0.4147, 95% confidence interval: −0.7119 to 0.008456, Coefficient of determination (*r* squared) = 0.1720, two-tailed *P* value is 0.0550). Correlating rCBF and ptiO2 during intra-arterial nimodipine infusion, we get high significance from 2 hours until day 4 with too little data on day 5 to get valuable statistic information. We have picked out “day one” to show correlation (correlation coefficient (*r*) = −0.7348, 95% confidence interval: −0.8829 to −0.4538, coefficient of determination (*r* squared) = 0.5400, two-tailed *P* value is <0.0001); see Figure 5.

Spearman rank test shows significant correlation for ptiO2 before treatment (Spearman *r* = 0.6667 (corrected for ties), 95% confidence interval: 0.3290 to 0.8532, two-tailed *P* value is 0.0007) and during continuous intra-arterial nimodipine therapy (Spearman *r* = 0.8008 (corrected for ties), 95% confidence interval: 0.5633 to 0.9160, two-tailed *P* value is <0.0001) with GOS on discharge.

Results of CT perfusion scanning are shown in Table 5. One can see from the data that initial perfusion scanning missed vasospasm only once and therefore seems to be a very sensitive monitoring parameter.

## 4. Discussion

Continuous selective intra-arterial infusion of nimodipine has been described before by Musahl et al. [20]. In contrast to our results, none of their patients had “new ischemic lesions caused by vasospasm” on discharge. This aspect is alarming, as ischemia is one of the main reasons for neurologic deficit in these patients. In our cohort, we had started continuous intra-arterial nimodipine on day 9.19 ± 3.31 after SAH. On average, Musahl started more than one day earlier: day 7.5 ± 2.06. Comparing these data, an important issue seems to be the onset of treatment.

In our cohort from thirty patients, two had incidental MCA bifurcation aneurysm and were treated with clipping. One patient had clinical symptoms of confusion and speech disturbance after awakening from surgery. CT perfusion showed reduced perfusion frontotemporal but no area of infarction. Symptoms disappeared within a few hours and, as a result, no angiography was performed. Five days later, motor and sensory speech deficits reoccurred. CT perfusion and native cranial CT (CCT) showed infarction in the median territory frontotemporal. On angiography, vasospasm of a distally from the clip localized MCA branch plus the anterior cerebral artery, segment 1 (A1) left-sided, showed vasospasm. After two days, symptoms improved and the catheter was removed. Under logopedic therapy, the patient was able to articulate and understand with only minor deficits. The other Hunt & Hess 0 case was admitted to our hospital because of transitory ischemic attack (TIA) with dysarthria and hemiparesis. On CT angiography, two aneurysms of the right median bifurcation were identified. Transcranial Doppler showed both-sided M1- and left-sided ICA stenosis and a free ICA on the right with a history of thromboendarterectomy (TEA). Both aneurysms were clipped. After anesthesia, epileptic seizures occurred. The perfusion scan showed hyperperfusion of the right hemisphere, especially parietooccipital. The patient did not wake up. CCT and CT perfusion seven days after clipping illustrated a swollen right hemisphere and hypodense brain tissue in the right-sided median and posterior territories, suspect for infarctions. After 3 days of continuous intra-arterial nimodipine infusion, the vasospasm had disappeared, but infarction areas were fixed and, with it, the patients symptoms on waking. On discharge, a left-sided hemiparesis and psychomotor deceleration were noticed.

All of our patients showed persistent vasospasm after 30 minutes of intra-arterial infusion of 10 mL nimodipine solution in the angiography unit. Vasospasm improved in 26 patients after continuous intra-arterial nimodipine infusion. Recurrence of vasospasm was seen in 4 patients who received the same treatment once more. A third cycle was only needed once. All patients who needed more than one treatment cycle of continuous intra-arterial nimodipine infusion were graded Fisher 4 on initial CT scan.

It is remarkable that ptiO2 values showed such prompt and extensive response. Value changes in other cases were not as high as reported by Musahl et al. [20]. Hoelper et al. [21] also have described the increase of ptiO2, but Stiefel et al. [22] have found decreased tissue oxygenation during endovascular vasospasm therapy.

Musahl et al. [20] describe the great effect on flow velocities in transcranial Doppler after onset of intra-arterial nimodipine treatment. In our study, we could not detect reliable changes in flow velocities. In some cases we missed clinical and angiographic improvement of vasospasm and in others we did not detect beginning or manifest vasospasm. A review by Lysakowski et al. [23] describes transcranial Doppler as not decisive and so should not be used as the only neuromonitoring parameter.

Our patients showed great response of rCBF-values on intra-arterial nimodipine administration. The same effect could be shown by Vajkoczy et al. [24], using intra-arterial papaverine for antivasospastic therapy.

In correlation with Moftakhar et al. [25] and Majoie et al. [26] results we found good correlation of vasospasm and findings in CT perfusion scanning. It marked tissue at risk with prolonged time to peak and mean transit time and showed a mismatch of cerebral blood flow and cerebral blood volume with a sensitivity of 97% in our case series. Native CAT scan missed many of these cases especially in early stage of vasospasm. Some of our patients never showed ischemic changes on native CT scan even if CT perfusion detected tissue at risk areas for over a week with correlating angiographic vasospasm.

Our protocol for continuous selective intra-arterial nimodipine infusion is easily feasible. Infrastructural prerequisites include angiography plus intensive or intermediate care unit. The radiologist should be experienced in the interpretation of intracranial digital subtraction angiography and be able to intervene. A neurosurgeon should at least be on call if major problems occur, which need to be treated surgically. Most hospitals treating patients with SAH dispose of these departments anyway. The equipment needed is a 4 or 5 French introducer sheath with a microcatheter + nimodipine solution. Multimodal neuromonitoring is recommended.

Vessel wall dissection, catheter dislocation, and embolic infarction due to thrombotic adhesion at catheter or vessel wall are risks which should be kept in mind but have not been reported so far. There were three suspect findings for thrombotic vessel wall adhesions on CT angiography, but none of these patients suffered embolic infarction. To prevent embolic complications, anticoagulation is an important issue. In our study, 10,000 IU/24 h heparin was infused via the same catheter as nimodipine. Mayer et al. [27] and Musahl et al. [20] used comparable protocols and did not notice serious side effects or complications.

From thirty patients, one died of sepsis three days after catheter removal. Even if the catheter tip was sterile on microbiological tests, we cannot rule out correlation between intra-arterial catheter and sepsis. But having in mind that most of these patients were critically ill a sepsis rate of 3.3% is within the normal range, even lower, than in other ICU series [28].

None of the dead patients were autopsied. Therefore, causes of death are clinical interpretations but cannot be assured. One could say that this issue is a “black box.” We could have missed death causing problems connected to the catheter. Risk analysis for that is not purposeful.

We could not detect correlation between patient age and onset of vasospasm, duration of intra-arterial nimodipine application, or outcome. Results were the same for correlation of sex and other parameters. Hunt & Hess did not predict duration of therapy, but the Spearman rank correlation test showed significant difference in outcome; see Figure 3. The onset of continuous intra-arterial nimodipine treatment significantly correlates with the outcome (GOS); see Figure 4.

Results in our study are worse than those reported by Musahl et al. [20]. Comparing the patient population in both studies, mean age is 47 years with Musahl et al. [20] and 49.8 years in our study. The WFNS compared to the Hunt & Hess score used in our study does not show a great difference in the severity of cases included in both studies. Two-thirds of Musahl et al. [20] patients were coiled on their aneurysm whereas only 7% of our patients were coiled as the only aneurysm treatment. One more aspect is the onset of intra-arterial nimodipine treatment. Musahl et al. [20] started one day earlier after bleeding and within 2 hours of onset of symptoms, which presupposes an excellent infrastructure. Patient age, aneurysm coiling, and time onset of treatment seem to be important facts for better patient outcome. The first cannot be influenced. The next two should be analyzed in further studies.

We only included patients that did not respond to triple H therapy plus short-time intra-arterial infusion of nimodipine in the angiography unit, which makes the patients included in our study have more severe vasospasm from the start.

## 5. Conclusion

As selective continuous intra-arterial nimodipine infusion seems to be a safe and easy therapy, one should think of implementing it as soon as possible to prevent permanent ischemia. Multimodal neuromonitoring seems to be helpful in judging efficacy and safety of this new established treatment. Further investigations could reveal certain criteria, in which patients are likely to profit from continuous intra-arterial nimodipine application and in whom a different therapy should be started. Up till now, patient cohorts are too small to give recommendations. This should change in the near future.

## Figures and Tables

**Table 1:** Clinical patient and treatment data.

Case no.	Age (years)	Sex	H & H grade	Fisher grade	Aneurysm localization	Treatm. ons. (day)	Treatm. dur. (days)	Outcome (GOS)	Coiling versus clipping
1	54	f	3	4	A com A	9	4	1	Clipping
2	42	f	5	4	MCA l	10	5	1	Clipping
3	43	f	5	4	MCA r	9	4	1	Clipping
4	42	m	3	3	MCA l + ICA l	5	2	5	Clipping
5	53	f	4	4	MCA r	8	4	5	Clipping
6	56	m	2	2	A com A	9	3	2	Clipping
7	43	f	3	4	MCA l	13	3	3	Clipping
8	29	m	5	3	P com A r	7	3	1	Clipping
9	48	f	2	2	A com A	5	3	4	Clipping
10	52	m	5	4	MCA r	14	2	2	Clipping + reclipping
11	48	f	3	4	MCA r + ICA r	6 + 10	3 + 3	5	Clipping + coiling
12	76	m	2	2	A com A	9	4	1	∖
13	50	f	5	4	MCA l	6	3	2	Clipping
14	56	f	2	3	A com A	8	3	4	Clipping
15	27	f	2	2	MCA l + carotid T l	7	3	5	Clipping
16	40	m	4	4	A com A	8 + 12	3 + 3	4	Clipping
17	68	f	2	2	ICA l	10	3	3	Clipping
18	55	f	4	4	C1/C2 r	9	3	3	Clipping
19	62	f	2	3	A com A	6	2	5	Clipping
20	48	m	0	1	MCA r	7	3	3	Clipping
21	49	m	5	4	ICA r	11	3	3	Clipping
22	66	m	4	4	MCA r	12	2	3	Clipping
23	41	f	2	3	MCA l	10	5	4	Clipping
24	61	f	3	4	MCA r	1 + 10	3 + 3	3	Clipping
25	48	m	0	1	MCA l	5	2	5	Clipping
26	47	f	1	4	A com A	7	3	5	Coiling
27	42	f	3	3	MCA r	11	2	3	Clipping
28	38	m	3	4	P com A l	2 + 8 + 13	3 + 4 + 4	5	Coiling
29	53	f	4	4	A com A	12	3	2	Clipping
30	56	f	4	4	MCA r	20	1	5	Clipping

Case no.: case number; H & H grade: Hunt and Hess grade; treatm. ons. (day): first day of selective continuous intra-arterial nimodipine infusion; treatm. dur. (days): duration of selective continuous intra-arterial nimodipine infusion in days; outcome (GOS): clinical outcome on discharge in Glasgow outcome scale score; coiling versus clipping: interventional neuroradiologic aneurysm coiling versus microsurgical aneurysm clipping; f: female; m: male; r: right; l: left; A com A: anterior communicating artery; MCA: median cerebral artery; ICA: internal carotid artery; P com A: posterior communicating artery; carotid T: bifurcation of internal carotid artery; C1/C2: segments of the internal carotid artery; ∖: no coiling or clipping.

**Table 2:** rCBF before, at 2 and 6 hours, and 1–5 days after starting continuous selective intra-arterial nimodipine infusion, rCBF (mL/100 g/min).

Case no.	rCBF before	rCBF at 2 hours	rCBF at 6 hours	rCBF day 1	rCBF day 2	rCBF day 3	rCBF day 4	rCBF day 5
1	14	22	24	23.3	17.6	9.5	6.3	0
2	17	61	66	44.7	34.8	24.3	7.3	4.2
3	17	46	48	47.3	45.8	36.3	37.5	0
4	16	61	63	50.1	44.4	0	0	0
5	19	58	54	45.6	38.3	33.7	35.1	0
6	12	38	34	33.1	28.4	19.0	0	0
7	13	46	46	43.8	36.2	33.4	0	0
8	12	49	44	23.0	19.3	12.5	0	0
9	17	47	50	48.7	43.9	38.3	0	0
10	16	23	25	22.3	14.5	0	0	0
11	16	73	68	53.8	49.7	45.7	0	0
12	14	24	20	23.7	22.6	12.2	9.3	0
13	12	40	33	30.4	24.0	22.2	0	0
16	15	53	54	45.3	39.3	37.2	0	0
18	8	57	50	38.4	30.6	20.4	0	0
21	12	46	44	29.6	22.9	17.5	0	0
22	7	40	39	35.2	33.8	0	0	0
23	15	55	51	44.8	41.6	37.9	33.9	35.6
24	16	60	54	46.2	42.9	26.7	0	0
27	14	43	44	36.2	22.3	0	0	0
28	12	45	43	36.7	36.8	39.4	0	0
29	9	24	34	23.0	15.7	13.5	0	0

rCBF: regional cerebral blood flow; 0: no continuous selective intra-arterial nimodipine application.

**Table 3:** ptiO2 before, 2 and 6 hours, and 1–5 days after starting continuous selective intra-arterial nimodipine infusion, ptiO2 (mmHg).

Case no.	ptiO2 before	ptiO2 at 2 hours	ptiO2 at 6 hours	ptiO2 day 1	ptiO2 day 2	ptiO2 day 3	ptiO2 day 4	ptiO2 day 5
1	9	21	20	15.4	10.9	4.3	3.7	0
2	4	20	19	19.2	19.4	14.3	4.8	2.9
3	14	22	23	19.3	21.5	22.3	22.4	0
4	14	27	26	23.4	23.5	0	0	0
5	15	29	26	26.3	26.8	24.2	27.9	0
6	9	18	17	17.5	18.3	12.2	0	0
7	11	14	16	16.3	17.2	17.5	0	0
8	6	15	16	14.2	9.3	4.2	0	0
9	12	19	22	22.3	24.8	24.7	0	0
10	10	18	17	17.9	18.0	0	0	0
11	14	22	25	23.4	22.9	22.6	0	0
12	5	16	17	14.3	14.9	7.9	6.6	0
13	8	20	22	23.6	22.8	22.0	0	0
16	13	21	24	22.4	21.8	22.9	0	0
18	5	18	19	21.1	18.3	18.6	0	0
21	11	20	19	19.3	20.4	18.3	0	0
22	8	18	18	19.5	17.3	0	0	0
23	10	23	22	24.7	24.4	28.7	30.5	29.3
24	14	20	22	20.3	12.3	9.8	0	0
27	14	21	21	22.3	19.5	0	0	0
28	14	25	24	24.3	24.0	25.6	0	0
29	7	17	16	16.9	18.3	16.2	0	0

ptiO2: partial tissue oxygenation; 0: no continuous selective intra-arterial nimodipine application.

**Table 4:** Transcranial Doppler mean flow values (cm/s) of the right- and left-sided median cerebral artery in six patients before and during continuous intra-arterial nimodipine therapy.

Case no.	Before i.a. nimodipine		Day 1		Day 2		Day 3		Day 4	
	Right	Left	Right	Left	Right	Left	Right	Left	Right	Left
1	184	156	190	183	174	199	168	186	193	205
4	115	94	110	114	89	74				
9	83	87	75	73	70	81	92	80		
10	204	220	164	156	128	140				
25	98	110	95	112	92	98				
30	90	86	73	71						

**Table 5:** Results of CT perfusion scanning before, during, and after treatment with continuous intra-arterial nimodipine infusion.

Case no.	Before		During		After
	Tissue at risk	Infarction	Tissue at risk	Infarction	Infarction
1	Yes	Minor	Yes	Major	Major
2	Yes	No	Yes	Major	Major
3	Yes	No	Yes	Minor	Minor
4	Yes	No	No	No	No
5	Yes	No	Yes	No	No
6	Yes	No	Yes	Minor	Major
7	Yes	Minor	Yes	Minor	Multiple minor
8	Yes	Major	Yes	Major	Major
9	Yes	No	Yes	No	Minor
10	Yes	No	Yes	Minor	Major
11	Yes	No	Yes	No	Minor
12	Yes	No	Yes	Major	Major
13	Yes	No	Yes	Minor	Major
14	Yes	Minor	Yes	No	Multiple minor
15	Yes	No	No	No	No
16	Yes	No	Yes	No	Minor
17	Yes	No	Yes	Minor	Multiple minor
18	Yes	Minor	Yes	No	Minor
19	Yes	No	Yes	No	Minor
20	Yes	No	Yes	Minor	Multiple minor
21	Yes	Minor	Yes	No	Multiple minor
22	Yes	No	Yes	No	Minor
23	Yes	No	Yes	No	Minor
24	Yes	No	Yes	Minor	Multiple minor
25	No	No	Yes	No	No
26	Yes	No	Yes	No	Minor
27	Yes	No	Yes	No	Minor
28	Yes	No	Yes	Minor	Minor
29	Yes	Minor	Yes	Major	Major
30	Yes	No	Yes	Minor	Minor (cerebellar)
